# Long-term oral nitrate therapy is associated with adverse outcome in diabetic patients following elective percutaneous coronary intervention

**DOI:** 10.1186/1475-2840-10-52

**Published:** 2011-06-13

**Authors:** Kai Hang Yiu, Vincent Pong, Chung Wah Siu, Chu Pak Lau, Hung Fat Tse

**Affiliations:** 1 Division of Cardiology, Department of Medicine, Queen Mary Hospital, The University of Hong Kong, Hong Kong

**Keywords:** Nitrate, Diabetes, MACEs

## Abstract

**Background:**

To assess the impact of long-term oral nitrate therapy on clinical outcome following percutaneous coronary intervention (PCI) in patients with type II diabetes.

**Methods:**

The incidence of major adverse cardiovascular events (MACEs) following elective PCI for stable coronary artery disease was evaluated in 108 patients with type II diabetes (age 64.6 ± 10.5 years, 67.7% men). Major adverse cardiovascular events were defined as the need for revascularization, non-fatal myocardial infarction or cardiovascular death. Multivariate Cox regression analysis was used to evaluate the predictive value of MACEs by clinical characteristics and the prescription of long-term nitrate therapy.

**Results:**

Isosorbide mononitrate (ISMN) was prescribed to 46 patients with an average dose of 44.3 ± 15.2 mg/day. After a mean follow up of 25.3 ± 25 months, 16 patients developed MACEs. Patients who received ISMN were more likely to suffer from MACEs (26.1% vs. 6.5%, P = 0.01), mainly driven by a higher rate of acute coronary syndrome (13.0 vs 0%, P = 0.01). Average daily dose of nitrate and other cardiovascular medication was not associated with MACEs. Multivariate Cox regression analysis revealed that prescription of only ISMN (Hazard Ratio 3.09, 95% CI 1.10-10.21, P = 0.04) was an independent predictor for the development of MACEs.

**Conclusion:**

Long-term oral nitrate therapy was associated with MACEs following elective coronary artery revascularization by PCI in patients with type II diabetes.

## Introduction

Elective percutaneous coronary intervention (PCI) is a common treatment for patients with stable coronary artery disease and comprises 85% of all PCI procedures [1,2]. Diabetic patients account for up to one quarter of patients who undergo PCI each year and experience a higher rate of post-operative adverse cardiovascular events than non-diabetics [3].

Organic nitrate remains one of the most frequently prescribed anti-anginal agents for the treatment of coronary artery disease (CAD), although no long-term beneficial effect has been proven [4]. Previous clinical trials have suggested that continuous administration of oral nitrates paradoxically increases adverse cardiac events following myocardial infarction [5-7]. It is nonetheless remains unknown whether the use of oral nitrates following elective PCI has a deleterious effect in patients with diabetes. The objective of this study was to determine the impact of long-term oral nitrate therapy on clinical outcome in patients with type II diabetes who undergo elective PCI for stable CAD.

## Methods

### Patients

Consecutive patients with type II diabetes and stable clinical symptoms who underwent successful elective PCI and coronary stenting for stable CAD between March 2003 and September 2005 were recruited. All patients had type II diabetes as defined by the American Diabetic Association [8], and were prescribed a hypoglycemic agent (oral antidiabetic agents or insulin). Patients were excluded if they had terminal malignancy, congestive heart failure, incomplete or failed revascularization (residual stenosis > 50% in any one of the three major coronary arteries), significant left main CAD > 50% stenosis, recent stroke or acute coronary syndrome in the past 3 months. There was no restriction in terms of usage of either bare metal or drug eluting stents.

### Study Design

Baseline clinical characteristics including body weight, height, and routine blood biochemistry were documented in all patients during their admission for PCI. Left ventricular ejection fraction (LVEF) was also evaluated by transthoracic echocardiography before PCI and patients were categorized as having preserved LVEF ≥50% or impaired LVEF < 50%. Data on medication prescribed before and after PCI were ascertained from the hospital computer system. Patients prescribed oral nitrate were given long release isosorbide-5-mononitrate (ISMN). All patients were followed up regularly in our clinic every 3-4 months. Data concerning all hospital admissions and death were retrieved from the hospital electronic record system. During the study period, no patients were lost to follow up. The presence of triple CAD was defined as the presence of lesions in all three major coronary arteries that were either successfully revascularized or had < 50% residual stenosis. This study was approved by the local institutional ethic committee.

The endpoint of this study was the occurrence of major adverse cardiovascular events (MACEs) including (1) the need for targeted vessel revascularization due to in-stent restenosis, or (2) non-fatal myocardial infarction, defined as the presence of symptoms consistent with the World Health Organization criteria [9], associated with abnormal levels of necrosis markers (including troponin) or diagnostic electrocardiogram changes, and (3) cardiovascular mortality (sudden cardiac death, fatal stroke, myocardial infarction and heart failure).

### Statistical Analysis

Continuous variables are presented as mean ± 1 standard deviation. Categorical data are presented as frequencies and percentages. Statistical comparisons were performed with Mann-Whitney U test or Chi-squared test, as appropriate. The association between clinical characteristics, underlying triple vessel disease and cardiac medication and the risk of MACEs was analyzed using Cox proportional hazards models. Multivariate analysis was performed with an enter regression model, in which each variable with a P value ≤ 0.1 (based on the univariate analysis) was entered into the model. Calculations were performed using SPSS software (version 15.0). A P value < 0.05 was considered statistically significant.

## Results

### Baseline demographics

A total of 280 patients underwent elective PCI and 108 patients who satisfied the inclusion criteria were followed up for a mean period of 25.3 ± 25 months. Their mean age was 64.6 ± 10.5 years and 67.6% were men. In total, 20 patients (18.5%) required insulin therapy and the mean HbA1c level was 7.8 ± 2.2%. The body mass index was 25.7 ± 3.8 kg/m^2^. Triple vessel disease was present in 14 patients (11.1%) and 34 (31.5%) had undergone previous percutaneous intervention. A total of 20 patients (18.5%) had impaired LVEF < 50%. Beta-blocker, statin and angiotensin converting enzyme inhibitor or angiotensin receptor blocker was prescribed to > 80% of patients.

A total of 46 patients received ISMN therapy, prescribed for at least 1 month prior to PCI. The mean duration of ISMN therapy before PCI was 258.2 ± 169.7 days and the total duration of ISMN therapy before and after PCI was 816.1 ± 691.7 days. The mean total ISMN dose received per patient was 38.6 ± 40.7 gm and the average daily dose was 44.3 ± 15.2 mg/day. The baseline demographics of patients with and without ISMN therapy are shown in Table 1. There were no significant differences in terms of age, conventional cardiovascular risk factors or use of concomitant medication (P > 0.05). Patients who received ISMN were nonetheless more likely to suffer from underlying triple vessel disease (23.9 vs. 4.8%, P = 0.01).

**Table 1:** Baseline characteristics between patients with and without ISMN

Variable	Without ISMN (n = 62)	With ISMN (n = 46)	P value
**Baseline Demographics**			

Age, years	64.3 ± 10.1	65.0 ± 11.1	0.75

Male, n (%)	42(67.7)	31(86.1)	1.0

Body mass index, kg/m^2^	26.3 ± 3.9	25.0 ± 3.6	0.09

Hypertension, n (%)	15(24.2)	5(10.9)	0.09

Smoking, n (%)	29(46.8)	23(50.0)	0.70

Insulin therapy, n (%)	13(21.0)	7(15.2)	0.62

Creatinine, mmol/L	141.1 ± 137.4	128.8 ± 91.9	0.59

HbA1c, %	7.8 ± 2.6	7.8 ± 1.4	0.92

Total cholesterol, mmol/L	4.4 ± 1.0	4.5 ± 1.3	0.88

Triglyceride, mmol/L	1.9 ± 1.6	2.1 ± 4.0	0.76

HDL cholesterol, mmol/L	1.1 ± 0.2	1.2 ± 0.2	0.18

LDL cholesterol, mmol/L	2.6 ± 0.9	2.5 ± .7	0.76

Triple vessel disease, n (%)	3(4.8)	11(23.9)	*0.01

Prior myocardial infarction, n (%)	15(24.2)	18(39.1)	0.14

Prior PCI, n (%)	17(27.4)	18(39.1)	0.21

Drug eluting stent, n (%)	43(69.4)	31(67.4)	0.84

Impaired LVEF < 50%, n(%)	13(21.0)	7(15.2)	0.62

**Medications after PCI**			

Beta blocker, n (%)	50(80.6)	38(82.6)	1.0

Calcium channel blocker, n (%)	43(69.4)	29(63.0)	0.40

Statin, n (%)	54(87.1)	41(89.1)	1.0

ACEI/ARB, n (%)	53(85.5)	39(84.8)	0.77

Aldosterone blocker, n (%)	6(9.7)	1(2.2)	0.14

Abbreviation: ACEI = Angiotensin converting enzyme inhibitor; ARB: Angiotensin receptor blocker; BMI = Body mass inex, HDL = High density lipoprotein; ISMN = Isosorbide-5-monitrate; LDL = Low density lipoprotein; LVEF = Left ventricular ejection fraction; PCI = Percutaneous coronary intervention;

*: P < 0.05

### Major adverse cardiovascular events

The clinical outcomes of the study population are summarized in Table 2. Overall, 16 patients (14.8%) developed MACEs and all-cause mortality was 4.6%. Among patients with MACEs, 7 developed in-stent restenosis that required repeat revascularization (presented with crescendo angina) and 6 were hospitalized for non-fatal myocardial infarction. Cardiovascular death occurred in 3 (sudden death in 1 patient and fatal stroke in 2). Patients who received ISMN were more likely to suffer from MACEs (26.1 vs. 6.5%, P < 0.01), mainly driven by a higher rate of non-fatal myocardial infarction (13 vs. 0% P < 0.01) (Table 2). There were no differences in occurrence of in-stent restenosis, cardiovascular mortality or all-cause mortality between patients with and without ISMN therapy.

**Table 2:** Duration of follow up and incidence of MACE in patients with and without ISMN

	All (n = 108 )	Without ISMN (n = 62)	With ISMN (n = 46 )	P value
Duration of Follow-up (months)	25.3 ± 25	26.2 ± 10.8	24.1 ± 13.5	0.39

MACE, n (%)	16 (14.8)	4 (6.5)	12 (26.1)	* < 0.01

Cardiovascular mortality, n (%)	3 (2.8)	2 (3.2)	1 (2.2)	1.00

Non-fatal myocardial infarction, n (%)	6 (5.5)	0 (0)	6 (13.0)	* < 0.01

In-stent restenosis, n (%)	7 (6.5)	2 (3.2)	5 (10.9)	0.13

All cause mortality, n (%)	5 (4.6)	3 (4.8)	2 (4.3)	1.00

Abbreviation: Similar to Table 1, MACE: Major adverse cardiovascular events.

**Figure 1:**
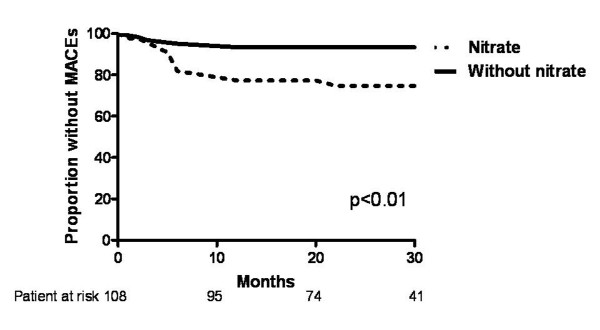
**Kaplan-Meier curve for free from major adverse cardiovascular events (MACEs) in patients with and without long-term oral nitrate therapy**.

**Figure 2:**
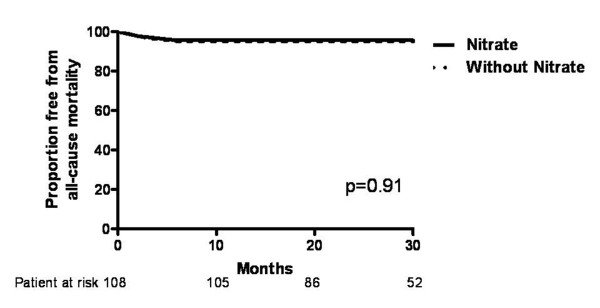
**Kaplan-Meier curve for free from all-cause mortality in patients with and without long-term oral nitrate therapy**.

The MACEs and all-cause mortality-free survival curves of patients with and without ISMN therapy are shown in Figure 1 and 2, respectively. During a follow up of over 40 months, the cumulative incidence of MACEs was significantly higher in patients with ISMN compared with those without (log-rank test P < 0.01), although all-cause mortality did not differ. The predictive value of clinical characteristics and medication on MACEs was analyzed by Cox proportional hazards analysis (Table 3). Univariate analysis revealed that age and ISMN were predictors of MACEs. Multivariate analysis demonstrated that only the use of ISMN independently predicted MACEs, not age, creatinine level or triple vessel disease.

**Table 3:** Factors predictive of MACE by Cox regression Model

	Univariate			Multivariate		
**Variable**	**HR**	**CI**	**P value**	**HR**	**CI**	**P value**

**Baseline characteristics**						

Age, years	1.03	1.01-1.10	0.02	1.01	0.96-1.06	0.81

Male	1.31	0.42-4.09	0.64			

BMI	0.92	0.81-1.05	0.23			

Hypertension	0.61	0.14-2.67	0.51			

Smoking	1.04	0.39-2.79	0.93			

Insulin therapy	1.61	0.37-7.12	0.53			

Creatinine	1.01	1.00-1.02	0.06	1.01	0.98-1.02	0.12

HbA1c	1.04	0.72-1.50	0.85			

Total cholesterol	0.84	0.45-1.57	0.59			

Triglyceride	0.93	0.63-1.39	0.73			

HDL	0.34	0.03-4.20	0.40			

LDL	0.90	0.40-1.98	0.79			

Triple vessel disease	3.03	0.83-11.07	0.09	1.37	0.16-11.51	0.77

Prior myocardial infarction	1.45	0.54-4.08	0.45			

Prior PCI	1.67	0.62-4.49	0.31			

Drug eluting stent	0.67	0.25-1.82	0.44			

Impaired LVEF < 50%	1.79	0.41-7.87	0.44			

**Medications**						

ISMN	3.88	1.25-12.0	0.02	3.09	1.19-10.21	0.04

Beta blocker	2.06	0.27-15.67	0.49			

CCB	1.58	0.45-5.59	0.48			

Statin	3.60	0.73-17.92	0.12			

ACEI/ARB	1.28	0.36-4.53	0.70			

Aldosterone blocker,	0.40	0.28-8.21	0.83			

Abbreviation similar to Tables 1 and 2.

## Discusssion

The present results demonstrate that ISMN therapy following elective PCI for stable CAD in patients with type II diabetes is associated with an increased risk of MACEs, mainly driven by an increased risk of non-fatal myocardial infarction. The average dose and total amount of ISMN exposure were nonetheless not associated with the likelihood of developing MACEs.

Previous large trials including the ISIS-4 [10] and GISSI-3 [11] trials have failed to demonstrate any beneficial effect of nitrate therapy in post myocardial infarction patients. A recent study of 1000 diabetic patients with acute myocardial infarction revealed that early revascularization and treatment with angiotensin converting enzyme inhibitors, angiotensin II receptor blocker, and aspirin, not nitrates, are associated with improved survival [12]. Previous retrospective studies have also demonstrated that chronic oral nitrate therapy may have a detrimental effect on the long-term outcome for patients with CAD [6,7]. In a study by Nakamura and colleagues, the use of oral nitrate therapy was associated with all-cause mortality in 2821 post myocardial infarction patients [7]. In another study of 1002 myocardial infarction patients randomly assigned to receive or not receive oral nitrate therapy, oral nitrate therapy was associated with adverse cardiac events (fatal or non-fatal recurrent myocardial infarction, congestive heart failure and sudden death). This adverse association has also been found in a prospective open label randomized trial in patients with healed myocardial infarction: patients who received oral nitrate therapy had a significantly higher rate of adverse cardiac events (6.6 vs. 3.1%, p < 0.05) after 102 months of follow up than those who did not [5]. Our results demonstrate that oral nitrate therapy in diabetic patients who undergo elective PCI is associated with adverse cardiovascular events, mainly driven by a higher incidence of non-fatal myocardial infarction. This raises concern about the potential adverse effects of oral nitrate therapy in patients with underlying ischemic heart disease, in particular those with type II diabetes. The recent advent of spinal cord stimulation and enhanced external counterpulsation may offer additional treatment options for patients with refractory angina [13].

Although the use of oral nitrate therapy may be associated with an adverse cardiovascular outcome, the underlying mechanism, particularly in diabetic patients, remains unclear. One of the major limitations of long-term oral nitrate treatment is the rapid diminution of its hemodynamic and anti-ischemic effects as a result of nitrate tolerance. Recent studies have also demonstrated that sustained nitrate therapy is associated with an increased production of reactive oxygen species (ROS) by uncoupling of endothelial nitrate oxide synthase and activation of protein kinase C [14-16]. In addition, hyperglycaemia may cause vascular damage that is mediated through an increased oxidative stress with the generation of ROS, such as superoxide (O_2_^-^), hydroxyl (OH) and peroxyl (RO_2_)[17-19]. The amplified oxidative stress in patients with type II diabetes and chronic nitrate therapy may aggravate the harmful vascular effects, and contribute to an increased adverse long-term clinical outcome. Another possible explanation of our findings is that the increased platelet aggregating activity in patients with CAD [20] and diabetes [21] following nitrate administration may subsequently lead to higher rates of cardiovascular events. The presence of diabetes and nitrate therapy has recently been shown to be associated with residual platelet reactivity in patients on clopidogrel therapy [22]. This evidence further supports our observation that chronic nitrate therapy may have a detrimental effect in type II diabetic patients with established CAD.

A limitation of our study was its retrospective nature: a randomized, placebo-controlled trial would be preferable. Prescription of medication was at the discretion of individual physicians and the rationale could not be easily qualified from patients' records. Although patients prescribed long term nitrate therapy were likely a group of patients with more angina symptoms (reflecting worse coronary disease), the independent predictive effect of MACEs persisted in such patients despite multivariate adjustment for underlying triple vessel disease. In addition, a significant proportion of diabetic patients may remain asymptomatic due to underlying silent myocardial ischemia. Thus symptom-driven prescription of oral nitrates may not allow accurate identification of diabetic patients with underlying myocardial ischemia. A study involving 310 diabetic patients who underwent coronary angiogram with or without revascularization has demonstrated that asymptomatic diabetic patients have a higher cardiac death risk (26.4 vs. 8.8%; *P *< 0.001) compared with well-matched symptomatic diabetic patients during a 5 year follow up [23]. We believe that diabetic patients using oral nitrate may not necessarily represent an excessive higher risk subgroup. Lastly, the small study population may not allow the identification of other potential variables (for example triple vessel disease) that could be associated with MACEs.

## Conclusion

The present study demonstrated that long-term oral nitrate therapy is associated with MACEs following elective PCI for stable coronary artery disease in type II diabetic patients. Although a causal relationship could not be established due to the retrospective design, we believe that our results provide the rationale for a future randomized trial to validate this observation.

## Competing interests

The authors declare that they have no competing interests.

## Authors' contributions

KH participated in the study's design, data collection, statistical analysis and final preparation of the manuscript. VP participated in the data collection of the study. CW and CP participated in the design of the study. HF participated in the study's design and final preparation of the manuscript. All authors read and approved the final manuscript.
